# Mediolateral oversizing influences pain, function, and flexion after TKA

**DOI:** 10.1007/s00167-013-2443-x

**Published:** 2013-02-12

**Authors:** Michel P. Bonnin, Axel Schmidt, Luca Basiglini, Nadine Bossard, Emmanuelle Dantony

**Affiliations:** 1 Centre Orthopédique Santy, 24 Av Paul Santy, 69008 Lyon, France; 2 Hospices Civils de Lyon, Service de Biostatistique, 69424 Lyon, France; 3 Université de Lyon; Université Lyon I, 69622 Villeurbanne, France; 4 Laboratoire Biostatistique Santé, CNRS; UMR 5558, 69495 Pierre-Bénite, France

**Keywords:** Total knee arthroplasty, Oversizing, Pain, Knee flexion

## Abstract

**Purpose:**

Manufacturers of total knee arthroplasty (TKA) have introduced narrower femurs to improve bone-implant fit. However, few studies have reported the clinical consequences of mediolateral oversizing. Our hypothesis was that component oversizing negatively influences the results after TKA.

**Methods:**

One hundred and twelve prospectively followed patients with 114 consecutive TKA (64 females and 50 males) were retrospectively assessed. The mean age of the patients was 72 years (range, 56 to 85 years). The dimensions of the femur and tibia were measured on a preoperative CT-scan and were compared with those of the implanted TKA. The influence of size variation on the clinical outcomes 1 year after surgery was assessed.

**Results:**

Mediolateral overhang was observed in at least one area in 66 % of the femurs (84 % in females and 54 % in males) and 61 % of the tibia (81 % in females and 40 % in males). Twenty-two patients presented no overhang in any area and 16 had overhang in all studied zones. The increase in the Pain and KOOS scores were 43 ± 21 and 36 ± 18 in the patients without overhang and 31 ± 19 and 25 ± 13 in patients with overhang (*p* = 0.033; *p* = 0.032). Knee flexion was 127° ± 7 and 121° ± 11, respectively. Regression and latent class analysis showed a significant negative correlation between overall oversizing and overall outcome.

**Conclusions:**

This study confirms that oversizing may lead to worse clinical results in TKA. The clinical consequences are that surgeons should pay attention not to oversize implants during implantation nd that oversizing should be ruled out in case of so called unexplained pain.

**Level of evidence:**

IV.

**Electronic supplementary material:**

The online version of this article (doi:10.1007/s00167-013-2443-x) contains supplementary material, which is available to authorized users.

## Introduction

Recent anatomical studies have shown that the size and shape of the femur and tibia at the knee vary significantly among individuals, most notably between males and females [3, 6, 24]. As a consequence, certain manufacturers of total knee arthroplasty (TKA) prostheses have increased their size range and introduced narrower femurs in an attempt to provide a better fit between the bone and implant and to prevent peripheral component overhang [7, 8, 14, 22, 23]. Oversizing the implant can theoretically compromise the clinical outcome by increasing tension and capsular/ligamentous friction on the implants. However, its actual clinical consequences have not been sufficiently studied. Mahoney et al. [36] showed that femoral component overhang increased the risk of residual pain after TKA, but the use of narrower, femoral implants did not always improve the results [14, 19, 35, 37, 51]. Whether these narrower implants are warranted remains under debate.

The objective of the present study was to assess the clinical consequences of femoral and tibial component overhang. The study aimed to quantify the association between mediolateral femoral and tibial sizing and clinical outcomes such as residual knee pain, function, and flexion. Our primary hypothesis was that component overhang in relation to the bone contours negatively influences the clinical result in terms of pain, function and joint range of motion. Our second hypothesis was that there is an oversizing threshold beyond which the negative effect is observed.

## Materials and methods

In order to test the hypotheses, a series of 255 consecutive patients undergoing primary TKA by a single surgeon between January, 2008 and June, 2009 were retrospectively analyzed. In our institution, a CT scan is performed as part of a systematic preoperative work-up for TKAs [6], and all our patients are prospectively followed. This study was designed to measure the size of the femur and the tibia on the preoperative CT scan and to compare these measurements with the size of the prosthesis implanted. We then sought to determine whether a relation existed between the size difference (under- or oversizing) and the result at 1 year postoperative, analyzed using the KOOS score and knee flexion.

Thirty-four patients in whom CT analysis of bony contours could be difficult were excluded from this study: patients with a history of previous knee surgery or fracture around the knee and patients who demonstrated a preoperative loss of full extension greater than 10°. Seventy-nine patients in whom functional evaluation could be biased, were also excluded: patients with inflammatory arthritis, patients older than 85 years, patients who had a postoperative complication necessitating revision, patients who had undergone surgical intervention of the contralateral knee less than a year before evaluation, or who had a medical event that prevented the functional assessment. A series of 142 patients was used for this study. All patients signed an informed consent form and the institution ethics committee authorized the study. Twenty-six patients were also excluded because of an incomplete preoperative or postoperative KOOS questionnaire and four patients because their CT scan could not be used due to artifacts.

In all, 114 knees (64 females and 50 males) in 112 patients were included in the study. The indication for TKA was medial compartment osteoarthritis in 80 knees, lateral compartment osteoarthritis in 16 knees, combined osteoarthritis in 8 knees, patellofemoral osteoarthritis in 6 knees, and spontaneous necrosis of the medial condyle in four knees. Demographic characteristics of the series are mentioned in Table 1.

**Table 1:** Preoperative demographic characteristics of the series

	Series		Males		Females		*p* value*
	Mean ± SD	Range	Mean ± SD	Range	Mean ± SD	Range	
Age (years)	72 ± 7	56–85	71 ± 7	56–85	72 ± 7	56–85	n.s.
Weight (kg)	81 ± 15	45–125	87 ± 15	62–125	76 ± 14	45–105	0.0001
Height (cm)	168 ± 10	144–194	175 ± 7	155–194	162 ± 7	144–178	0.0001

* Between Females and males (Student *T* test)

*n.s.* *p* > 0.05

**Fig. 1:**
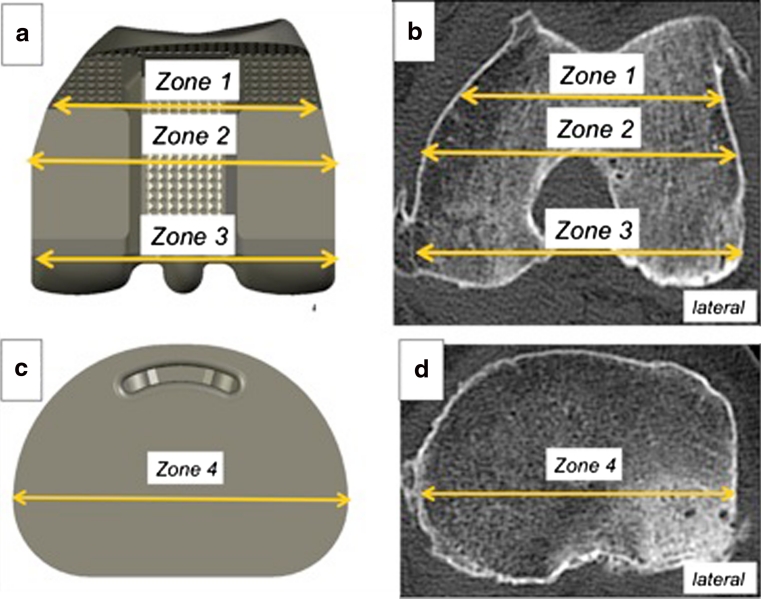
Three reference zones were defined on the femoral implant **a** zone 1, corresponding to the posterior part of the anterior chamfer, was located at a variable distance from the posterior bicondylar line (BCL) depending on the implant size (39.4–48.5 mm; see Appendix in ESM). Zone 2 was located at a variable distance from the posterior bicondylar line (BCL) depending on the implant size (26–36 mm; see Appendix in ESM), but was directly posterior to the point where the implant began to narrow. Zone 3 corresponds to the posterior condylar bone cut, situated 10 mm from the BCL. On the CT scan **b**, the analysis was done on the axial cut located at the level of the distal femoral cut made at the time of surgery (10 mm from the most distal point of the medial condyle). The bone dimensions corresponding to the three zones defined were measured: zone 1, 10 mm from the BCL, zone 2 and zone 3, at the distance corresponding to the size of the implanted prosthesis. On the tibia, the mediolateral dimension (zone 4) was used as the reference (**c**). On the CT scan, the measurement was taken on the axial cut located at the bone cut made at the time of surgery (**d**). The transverse, mediolateral dimension was measured

CT scan has been routinely performed as part of a systematic preoperative work-up for patients set to undergo TKA, in order to optimize rotational alignment of the femoral component with the transepicondylar axis. The CT scans were taken using a 64-slice multidetector scanner (Siemens^*®*^ Sensation, Munich, Germany). The measurements were taken by an experienced operator (AS), using OsiriX software with a technique that has previously been described [6]. For each knee, the mediolateral diameter was measured in three zones on the femur and in one zone on the tibia (Fig. 1). The measurements were taken at the level of the tibial cut and at the level the distal femoral cut made during the operation, which was documented in the surgical report. Each of these dimensions was compared to the corresponding dimension of the prosthetic component provided by the manufacturer (see Appendix in ESM). The difference between the preoperative and postoperative dimensions (“size variation”) was deemed positive in cases of implant oversizing and negative in cases of undersizing. Dimensions were measured in millimeters, with one decimal. For each dimension, the cortex was included in the measurement. A special attention was paid not to include osteophytes in the measurements. We defined oversizing as a difference greater than 0 mm. To assess the accuracy of the measurements we (MB, AS and LB) blindly repeated the measurements on twenty sets of CT-scans. A high level of intra and inter-observer reliability with errors of the mean always less than 1.5 mm was found.

Before surgery, each patient completed a KOOS functional assessment self-questionnaire at home in its validated French version [42]. After surgery, the patient completed a new KOOS self-questionnaire at home 1 year after the TKA. A follow-up visit 1 year after surgery was conducted by the senior rehabilitation physician, who was blinded to the size study. Maximum passive flexion (MPF) of the knee was measured at this time using a goniometer on the patient seated at the end of the examination table [52].

The prosthesis used was a posterior, stabilized implant with a fixed, tibial tray (HLS-Noetos, Tornier SA, Montbonnot, France, FDA approved device), which included six sizes and whose femoral and tibial aspect-ratio was close to other currently used implants (see Appendix in ESM) [6, 24]. All the prostheses were implanted using the same technique. Specifically, a medial parapatellar approach was used to evert the patella. The tibia was cut first, followed by the femur with a posterior reference. The tibial and femoral cuts were orthogonal to the mechanical axis so as to obtain a 180° axis. Rotation of the femoral component was aligned along the surgical transepicondylar axis, localized on the preoperative CT scan for each patient. Rotation of the tibial component was aligned with respect to the center of the anterior tibial tuberosity. The size of the components was determined based on the instrumentation so as to prevent any notch from being created along the anterior femoral cortex. The patella was resurfaced in such a way as to reproduce the preoperative patellar thickness. All the components were cemented (CMW3, DePuy, Warsaw, IN, USA). The same rehabilitation protocol was followed for all patients [5].

### Statistical analysis

The difference of oversizing between men and women was tested using a Student *T* test. The effect of size variation (under- or oversizing) in the four zones defined was analyzed with respect to pain, function, and flexion 1 year after implantation. To limit the risk of error related to multiplicity of statistical tests, three main variables were studied: pain was assessed using the pain subscore (*P*) of the KOOS score, overall function by the overall KOOS score, and flexion by the angle of MPF [33, 41]. For each patient, both the postoperative score and the score improvement were studied. The KOOS subscore values are presented in the Appendix of ESM. The analysis was carried out in four steps: (1) for each zone studied, two groups were compared: the oversized prosthesis group (size variation ≥0 mm) versus the normal or undersized prosthesis group (size variation <0 mm) using the unilateral, nonparametric Mann–Whitney test. Additionally, we compared the subgroups of patients in whom each zone was oversized versus those without any oversized zone. (2) The nonlinearity of the relationship was tested using smoothing splines and fractional polynomials [49]. To test nonlinearity, a *F* test was used based on an analysis of deviance between the models in which sizing was introduced linearly and the model in which sizing was introduced nonlinearly (degree of freedom = 4). (3) Linear regression models were then used to test the relation between MPF, increase of pain score or increase of KOOS score and size variation. (4) Finally, a multivariate and latent-class analysis was performed [4]. This analysis included four observed variables (size variation in the four defined zones) that reflected a latent variable representing the global “prosthetic fit”, and two other observed variables (pain score and flexion) that reflected another latent variable representing the global “post-operative outcome”. The relationship between the two latent variables was explored through a Spearman correlation. All analyses were performed using R software (latent class analysis was performed using the sem package from R software).

## Results

**Fig. 2:**
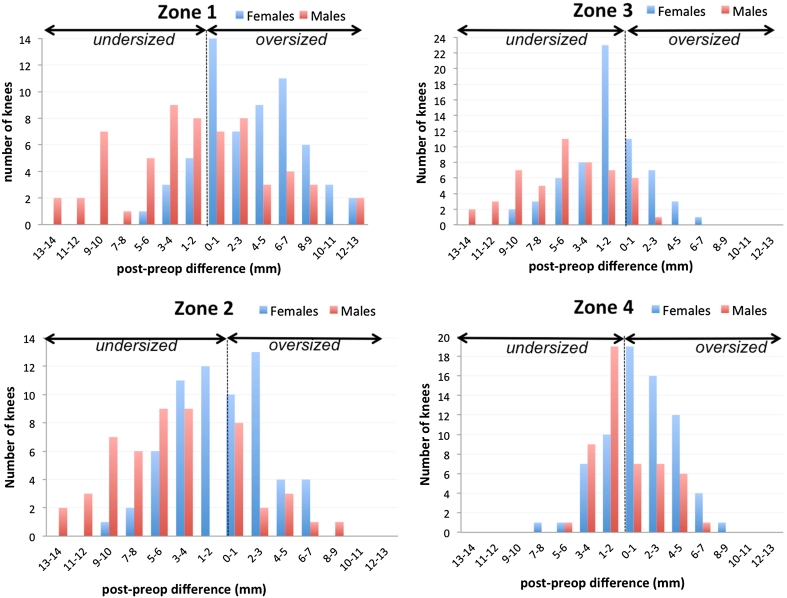
These *histograms* represent the distribution of the size variation (*X axis*) in the four zones studied in females (*blue columns*) and males (*red columns*)

In the femur, a medial–lateral prosthesis overhang greater than 0 mm was observed in 66 % of the knees in zone 1 (76 knees), 30 % in zone 2 (34 knees), and 23 % in zone 3 (26 knees). This proportion was 84, 48, and 34 % in females and 54, 30, and 14 % in males, respectively. For the tibia, medial–lateral overhang was found in 61 % (70 knees), 81 % in females and 40 % in males. Only twenty-two patients (18 men, 4 women) presented no overhang in any area and 16 had overhang in all zones (3 men, 13 women). For all the sizes studied, oversizing was significantly greater in females (Fig. 2; Table 2).

**Table 2:** Difference between preoperative dimensions measured on CT-scan and implant dimensions, for each of the four studied zones

	Series		Males		Females		*p* value*
	Mean ± SD	Range	Mean ± SD	Range	Mean ± SD	Range	
Zone 1	2.2 ± 5	−10/13	0.7 ± 5	−7/11	3.3 ± 4	−10/13	n.s.
Zone 2	−2.2 ± 5	−16/8	−4.2 ± 5	−16/8	−0.7 ± 4	−10/7	<0.001
Zone 3	−3.2 ± 4	−16/7	−5.4 ± 4	−16/2	−1.5 ± 3	−12/7	<0.001
Zone 4	0.9 ± 3	−7/8	−0.3 ± 3	−6/6	1.9 ± 3	−7/8	<0.001

* Between Females and males (Student *T* test)

Negative value means undersizing

*n.s.* *p* > 0.05

Preoperatively, women had a significantly lower flexion than males and lower Pain score, but preoperative KOOS score was not significantly different between males and females (Table 3). 1 year after surgery, pain score, KOOS score and knee flexion were lower in females. The gain of KOOS score was significantly lower in females but the gain of pain score was not significantly different (Table 4).

**Table 3:** Preoperative data

	Series		Males		Females		*p* value*
	Mean ± SD	Range	Mean ± SD	Range	Mean ± SD	Range	
Pain score	45 ± 15	0–94	49 ± 15	8–94	42 ± 15	0–69	0.030
KOOS score	36 ± 11	6–81	38 ± 12	12–81	34 ± 11	6–55	n.s.
Flexion (°)	105 ± 10	60/125	107 ± 8	60/125	102 ± 10	60/120	0.040
FTA (°)	176 ± 5	160–194	175 ± 5	165–186	177 ± 6	160–194	0.003

FTA: Femorotibial angle measured on the long leg X-Rays from the mediazl side (<180° means varus deformity)

* Between Females and males (Student *T* test)

*n.s.* *p* > 0.05

**Table 4:** Postoperative data

	Series		Males		Females		*p* value*
	Mean ± SD	Range	Mean ± SD	Range	Mean ± SD	Range	
Pain score	79 ± 18	28/100	84 ± 17	28/100	75 ± 18	36/100	0.005
KOOS score	64 ± 17	24/98	71 ± 17	31/98	59 ± 16	24/97	<0.001
Flexion (°)	122 ± 10	95/140	125 ± 8	100/140	121 ± 11	95/140	0.038
FTA (°)	178.2 ± 3	172/186	177.4 ± 3	172/183	178.9 ± 3	173/190	0.028
Increase in pain score	34 ± 19	−14/83	35 ± 19	−11/75	32 ± 19	−14/83	n.s.
Increase in KOOS score	29 ± 16	−16/68	33 ± 17	−6/68	25 ± 15	−16/57	0.018

FTA: Femorotibial angle measured on the long leg X-Rays from the medial side (<180° means varus deformity)

* Between Females and males (Student *T* test)

*n.s.* *p* > 0.05

Oversized patients in zone 1 had significantly lower pain score at follow-up compared with undersized patients and showed less improvement in the pain score. Patients with oversizing in zone 3 showed less improvement in the KOOS score at follow-up and had significantly lower postoperative flexion. Oversized patients in zone 4 had significantly lower postoperative flexion (Tables 5, 6).

**Table 5:** Effect of size variation in each zone on postoperative pain score, KOOS score, and knee flexion

Groups comparison					
	Under-sized		Over-sized		*p* value
	Mean ± SD	(Range)	Mean ± SD	(Range)	
Pain score					
Zone 1	82.5 ± 17.4	(27.8–100)	76.9 ± 18.1	(36.1–100)	0.034
Zone 2	79.8 ± 18.7	(27.8–100)	76.3 ± 16.4	(38.9–100)	n.s.
Zone 3	79.5 ± 18.7	(27.8–100)	76.2 ± 15.8	(44.4–100)	n.s.
Zone 4	81.1 ± 18.4	(36.1–100)	77.3 ± 17.8	(27.8–100)	n.s.
KOOS score					
Zone 1	67.6 ± 18.0	(31.3–97.0)	62.8 ± 16.7	(24.3–97.9)	n.s.
Zone 2	65.5 ± 17.5	(24.3–97.9)	61.7 ± 16.7	(25.0–94.1)	n.s.
Zone 3	64.8 ± 17.8	(24.3–97.9)	62.9 ± 15.4	(32.6–94.1)	n.s.
Zone 4	67.7 ± 16.8	(32.6–97.9)	62.3 ± 17.3	(24.3–97.0)	n.s.
Knee flexion					
Zone 1	124.6 ± 8.3	(105–135)	121.3 ± 10.4	(95–140)	n.s.
Zone 2	123.2 ± 9.1	(95–140)	120.6 ± 11.2	(100–140)	n.s.
Zone 3	123.4 ± 9.3	(95–140)	119.0 ± 11.0	(100–135)	0.038
Zone 4	124.7 ± 8.6	(100–140)	121.0 ± 10.3	(95–140)	0.034

*n.s.* *p* > 0.05

**Table 6:** Effect of size variation in each zone on the increase in pain score and KOOS score

Groups comparison					
	Under-sized		Over-sized		*p* value
	Mean ± SD	(Range)	Mean ± SD	(Range)	
Gain on pain score					
Zone 1	40.1 ± 20.0	(−11.1 to 83.3)	30.2 ± 18.1	(−13.9 to 75.0)	0.005
Zone 2	35.2 ± 19.8	(−11.1 to 83.3)	29.5 ± 17.6	(−13.9 to 75.0)	n.s.
Zone 3	34.8 ± 19.4	(−13.9 to 83.3)	29.1 ± 18.3	(−5.6 to 75.0)	n.s.
Zone 4	37.1 ± 21.7	(−5.6 to 83.3)	31.3 ± 17.4	(−13.9 to 75.0)	n.s.
Gain on KOOS score					
Zone 1	33.1 ± 18.9	(−4.9 to 68.3)	26.6 ± 14.8	(−16.0 to 61.2)	n.s.
Zone 2	30.3 ± 16.8	(−6.4 to 68.3)	25.1 ± 15.2	(−16.0 to 54.1)	n.s.
Zone 3	30.0 ± 16.8	(−16.0 to 68.3)	24.5 ± 14.8	(−5.9 to 61.9)	0.032
Zone 4	31.3 ± 18.5	(−5.9 to 68.3)	27.2 ± 15.0	(−16.0 to 59.6)	n.s.

*n.s.* *p* > 0.05

The increase in the pain score was 43 ± 21 in the group with no overhang in any zone (22 patients) and 31 ± 19 in the group with overhang in each of the four zones studied (16 patients) (*p* = 0.033). For the KOOS score, this gain was 36 ± 18 and 25 ± 13 respectively (*p* = 0.032). Mean flexion was 127° ± 7 in patients who presented no oversized zone and 121° ± 11 in those who were oversized in each of the four zones (ns).

**Fig. 3:**
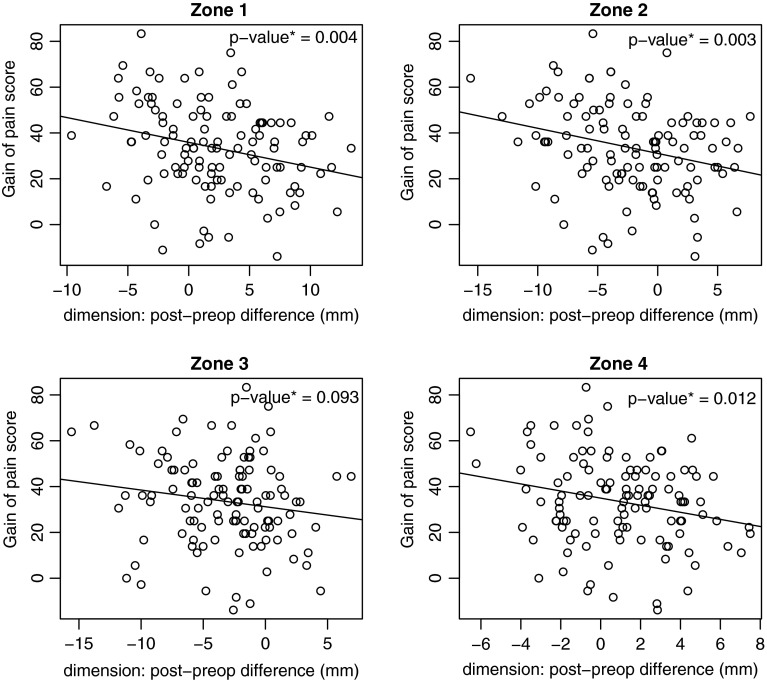
These figures represent the increase in the pain score (*Y axis*) in relation to the size variation (*X axis*) for the four zones studied. No threshold value was found on these *curves*

**Fig. 4:**
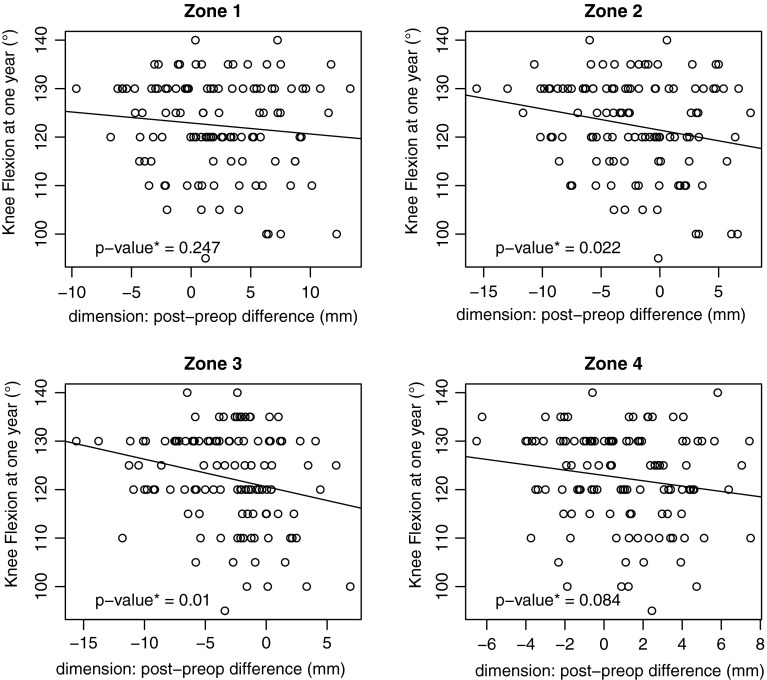
These figures show the flexion angle (*Y axis*) in relationship to the size variation (*X axis*) for the four zones studied. No threshold value was found on these *curves*

Results of the linear regressions demonstrated less improvement in the pain score and decreased knee flexion with oversizing. This relationship was significant for the pain score in zone 1 (*p* = 0.004), zone 2 (*p* = 0.003) and zone 4 (*p* = 0.012) (Fig. 3). For knee flexion, it was significant in zones 2 (*p* = 0.022) and zone 3 (*p* = 0.010) (Fig. 4). Globally, no nonlinear relationship was found and no threshold was observed.

**Fig. 5:**
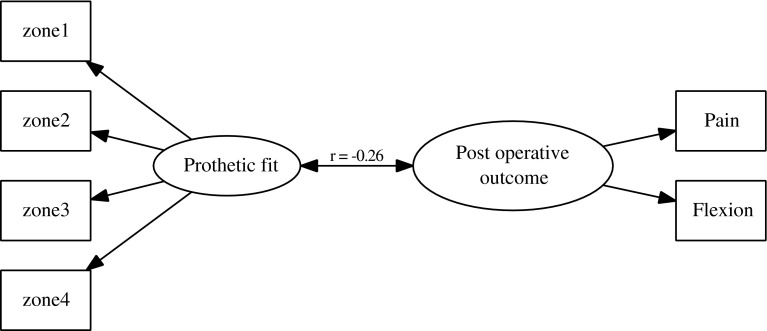
In the Latent Class Analysis, the first latent variable was defined as the “prosthetic fit”. It was obtained with the structural equation model from the measured variation of size in the four defined zones. The second latent variable was defined as the “post-operative outcome”. It was obtained with the structural equation model from the postoperative pain score and the MPF. The relationship between the two latent variables was explored through a Spearman correlation. In this structural equation model, the *rectangles* represent the observed variables while the *circles* represent the latent variables. The two latent variables, “prosthetic fit” and “post-operative outcome”, were found to be negatively correlated (*r* = −0.26 with a *p* = 0.005)

Using a structural equation model, the two latent variables “prosthetic fit” and “post-operative outcome” were found to be negatively correlated (*r* = −0.26, *p* = 0.005) (Fig. 5). When the value of the prosthetic fit was high (i.e. oversizing), the value of the postoperative outcome variable was low (i.e. a less favorable outcome).

## Discussion

The most important findings of the present study were that mediolateral oversizing encountered with commonly used implants was particularly frequent, particularly in women and that oversizing, whether in the femoral or tibial component, appears to lead to an increase in the rate of residual pain, poorer knee flexion, and a decreased overall functional result.

The strength of this study resides in the use of CT measurements, which are more precise than intraoperative measurements as described by Mahoney et al. [36]. Such precision allows quantifying both under- and oversizing in millimetric increments. In addition, the administration of a validated questionnaire filled out by patients at home, prevented investigator bias. Finally, The latent class analysis permitted to reinforce the global result indicating a correlation between sizing and functional outcome.

Certain limitations of this study should be noted. First, given that only a single implant was assessed, the observations made may not necessarily apply to other prostheses, even if the aspect ratio of the design used is close to that of other, more widely utilized implants (see Appendix in ESM). Second, the study was largely retrospective in nature, even if data were obtained from a prospectively followed series. Third, the exclusion of patients due to inadequate CT scans may have introduced selection bias. Similarly, the exclusion of patients that did not answer certain questions of the KOOS might introduce similar bias. Fourth, the study purposely only assessed the mediolateral dimensions given that the anteroposterior size variations influence the ligament balance and depend also on femoral rotation [21, 26, 34]. Finally, the measurements did not analyze separately medial or lateral overhang. It is possible that medial and lateral overhang have different clinical consequences.

An attempt to precisely match implants with the bony contours of the knee is sought during TKA. The consequences of poor fitting have previously been analyzed in the anteroposterior dimension: femoral oversizing can cause pain or stiffness [16, 31] and undersizing can lead to laxity [43, 44, 50], limitation of flexion [2], or anterior cortical notching [28, 38, 39, 53]. Few studies have assessed the consequences of mediolateral over- or undersizing [36]. The objective of the present study was to analyze the effect of mediolateral over- or undersizing of either the femoral or tibial component on function, residual pain, and flexion of the knee.

For each outcome criteria and each zone analyzed individually, the influence of sizing appeared to be moderate in our series. Indeed, when considering all zones, the maximal gains observed for pain score and KOOS score between undersized and oversized patients were 10 units and 6.5 units respectively and the difference regarding knee flexion at 1 year between these two groups did not exceed 4.5° (Tables 5, 6). However, oversizing occurred generally in multiple zones and outcomes were significantly lower in patients with multiple oversizing. Also, the latent class analysis showed a strong association between the global prosthesis oversizing and the global clinical outcome. Our results confirm the work of Mahoney et al. [36] who observed a twofold-increased risk of residual pain in cases of overhang of the femoral component greater than 3 mm. In our series, we did not observe such a strong relation but our definition of oversizing was a pre post-operative difference greater than 0 mm. For unicompartmental medial implants, Clarius et al. [13] reported medial tibial overhang greater than 2 mm in 45 % of the cases, but did not find a correlation with residual pain or the final functional result.

This study shows a surprisingly high rate of oversizing although non-oversizing was a priority during implantation. This can be explained firstly by the design of the implant, which is generally oversized in zone 1 but undersized in zones 2 and 3, secondly by the surgical technique; With the posterior referencing technique used in this series, the surgeon was sometimes obliged to accept an oversized implant in the ML dimension in order to avoid notching on the anterior cortex. Lastly, the limitation in the modularity, (i.e. femur size n cannot be used with a tibia size *n*−1 in the fixed bearing version of that prosthesis), forced sometimes the surgeon to make a compromise in the ideal sizing. However, it is interesting to note that Mahoney et al. reports similar findings, 76 % of his patients having an overhang >0 mm in at least one zone and 40 % of men and 68 % of women having an overhang ≥3 mm in at least one zone. Optimal sizing of the tibial component can also be challenging with “standard” implants due to the asymmetry of the native tibial plateaus, to the rotational landmark used in this series (alignment with the ATT) [6] and to the lack of modularity pushing the surgeon to use oversized tibia in order to match the femoral size. The popliteal tendon, semimembranous, and medialcollateral ligament are few anatomical structures, which may cause pain and decreased ROM with oversized tibial implants in the ML plane.

In the present series, preoperative knee flexion and pain scores were lower in females, which is consistent with data from other studies [9, 10, 17, 35, 45]. One year after surgery, the pain score, the KOOS score and knee flexion were still significantly lower in females compared to males and the increase in KOOS score was significantly higher in males. These data suggest that the results of TKA are worse in females, almost at one year follow-up, which confirms results reported by Ritter et al. [47] and Singh et al. [51]. However similar results between male and females have been also reported in other studies [9, 10, 18, 20, 27, 29, 30, 48] and this led some authors to challenge the principle of designing more narrow prostheses [1, 3, 35, 37]. Variations of the geometry of both the femur and tibia have been described and have been related to several factors including patient gender [3, 11, 12, 15, 24, 32, 35, 40, 46] but also morphotype [3] and ethnicity [25].

Surprisingly, the influence of size variation on clinical results was consistently linear and we observed no threshold effect. We therefore cannot determine an ideal implant size based upon the data, but can state the importance of not oversizing the components. Implant undersizing could theoretically be harmful by leaving an uncovered cancellous bone surface, where friction of the soft tissues on the bone ridges can cause pain [24]. Finally, the use of implants that are too small can be also a source of knee instability [42, 43, 50]. We did not demonstrate a negative effect of undersized implants on clinical outcomes. In fact, if anything it seemed to have a beneficial effect. This observation can perhaps be explained by our definition of under/oversizing, taking into account the ridge of the CT slice used. Due to the design of the borders of the femoral components, a normo-sizing according to our definition can be in fact an oversizing. Optimal sizing should be probably better analyze through volume imaging than surface imaging. This point warrants further investigation, but may have possible consequences on the design of these knee implants.

## Conclusion

This study confirms that mediolateral oversizing is a factor that may predict poor results in TKA. The findings also suggest that it is difficult to obtain optimal fit between the implant and bone in a large number of patients. The clinical consequences of this study are that surgeons should pay attention not to oversize implants during implantation and that oversizing should be ruled out in case of so called unexplained pain.

## Electronic supplementary material

Below is the link to the electronic supplementary material.

Supplementary material 1 (PDF 305 kb)
