# Supplementary material for: Mediolateral oversizing influences pain, function, and flexion after TKA
Source: Knee Surg Sports Traumatol Arthrosc. 2013 Feb 12;21(10):2314–24. doi: 10.1007/s00167-013-2443-x (PMC3777155; doi:10.1007/s00167-013-2443-x)
Supplement: Supplementary file 1 — Supplementary material 1 (PDF 305 kb) [file 167_2013_2443_MOESM1_ESM.pdf]

APPENDIX

SIZE AND ASPECT RATIO OF THE HLS-NOETOS TKA

**Dimensions of the HLS-Noetos components (mm)<br>(from Tornier SA, Montbonnot, France)**									
	**FEMUR**							**TIBIA**	
**Size**	**Zone 1**	**Zone 2**	**Zone 3**	**D^2**	**D^3**	**AP medial**	**AP lateral**	**Zone 4**	**AP**
1	52.57	60.7	60.7	16.23	29.4	52.9	54.1	37.3	39.9
2	57.82	64.24	64.24	17.76	30.9	56.2	57.6	40.4	42.9
3	61.21	68	68	20,38	32.6	60.4	61.9	43.6	45.5
4	64.46	71.97	71.97	22.16	35.3	63.9	65.4	46.6	48.1
5	69.26	76.18	76.18	24.04	36.6	67.5	69.2	49.8	50.7
6	73.82	80.63	80.63	26.03	38.5	71.3	73.2	52.9	53.7

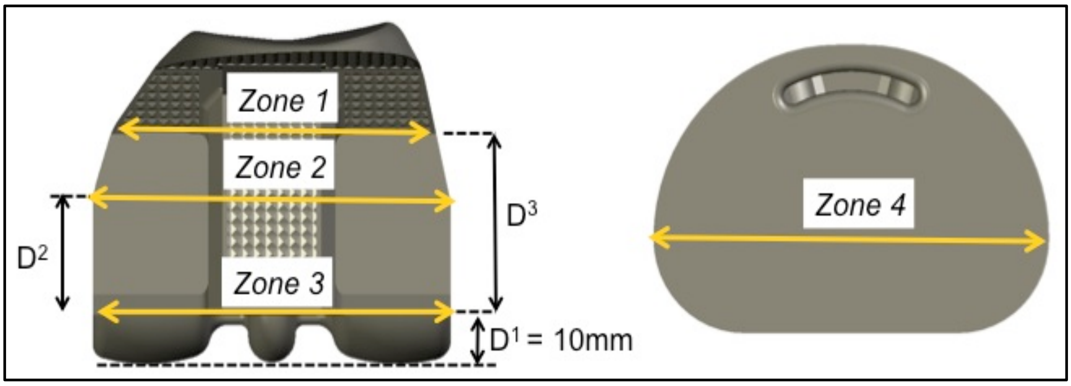

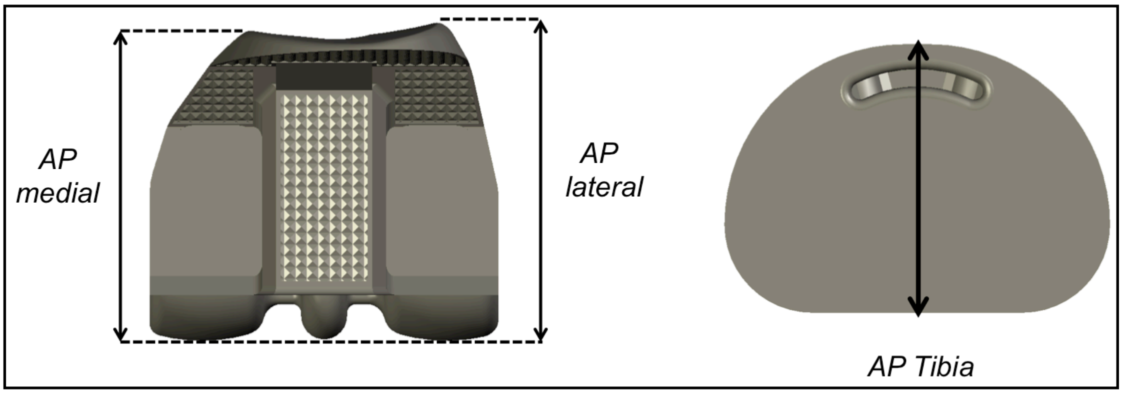

KOOS subscores in this series

KOOS Sub scores		Series Mean±SD	Series Range	Males Mean±SD	Males Range	Females Mean±SD	Females Range	p-value *
Before surgery	Symptoms	47±14	18/86	47±15	18/86	47±13	21/79	0.917
	Pain	45±15	0/94	49±15	8/94	42±15	0/69	0.030
	ADL	45±18	0/93	49±19	4/93	41±15	0/87	0.019
	QoL	25±15	0/81	25±14	0/81	25±16	0/75	0.994
	Sport	16±19	0/100	20±16	0/70	13±21	0/100	0.051
One year after surgery	Symptoms	63±16	18/100	63±18	18/100	63±14	32/100	0.826
	Pain	79±18	28/100	84±17	28/100	75±18	36/100	0.005
	ADL	76±19	22/100	81±18	44/100	72±20	22/100	0.008
	QoL	67±27	0/100	72±27	19/100	62±26	0/100	0.043
	Sport	38±32	0/100	54±30	0/100	25±27	0/100	<0.001
Increase in subscores	Symptoms	16±19	−29/68	16±21	−25/64	15±18	−29/68	0.800
	Pain	34±19	−14/83	35±19	−11/75	32±19	−14/83	0.38
	ADL	31±20	−25/78	32±21	−11/78	31±19	−25/68	0.709
	QoL	41±26	−19/94	46±28	−19/94	37±24	−13/88	0.068
	Sport	21±33	−75/100	34±35	−50/100	12±28	−75/80	0.001

* Between Females and males (Student T test)
